# Auxin-mediated regulation of vascular patterning in *Arabidopsis thaliana* leaves

**DOI:** 10.1007/s00299-018-2319-0

**Published:** 2018-07-10

**Authors:** Magdalena Biedroń, Alicja Banasiak

**Affiliations:** Department of Plant Developmental Biology, Institute of Experimental Biology, University of Wrocław, ul. Kanonia 6/8, 50-328 Wrocław, Poland

**Keywords:** Arabidopsis, Polar auxin transport, Leaf, Vascular system, Venation pattern, PIN

## Abstract

The vascular system develops in response to auxin flow as continuous strands of conducting tissues arranged in regular spatial patterns. However, a mechanism governing their regular and repetitive formation remains to be fully elucidated. A model system for studying the vascular pattern formation is the process of leaf vascularization in Arabidopsis. In this paper, we present current knowledge of important factors and their interactions in this process. Additionally, we propose the sequence of events leading to the emergence of continuous vascular strands and point to significant problems that need to be resolved in the future to gain a better understanding of the regulation of the vascular pattern development.

## Introduction

The development of the continuous conductive system is one of the most important morphogenetic processes occurring in plants. Proper construction and functioning of this system are necessary for the transport of water and nutrients, as well as the movement of signaling molecules (Dengler 2006; Lucas et al. 2013). Vascular strands are arranged in regular spatial patterns that enable all regions of the plant to be connected and integrated, but the mechanisms responsible for the formation of vascular spatial patterning are not yet fully understood.

Current knowledge about the process of vascularization is based mainly on studies of the venation pattern formation in the first vegetative leaf of Arabidopsis, one of the main models for research on vascular tissue development (for review see: Sawchuk et al. 2008; Donner and Scarpella 2009; Sack and Scoffoni 2013). In this review, we present the latest findings about the main factors and their interactions that are likely to be involved in the formation of the venation pattern. At the end, we propose the sequence of events leading to the emergence of this pattern in developing leaves and highlight the important issues that need to be resolved in the future.

## Venation pattern of Arabidopsis leaf

**Fig. 1:**
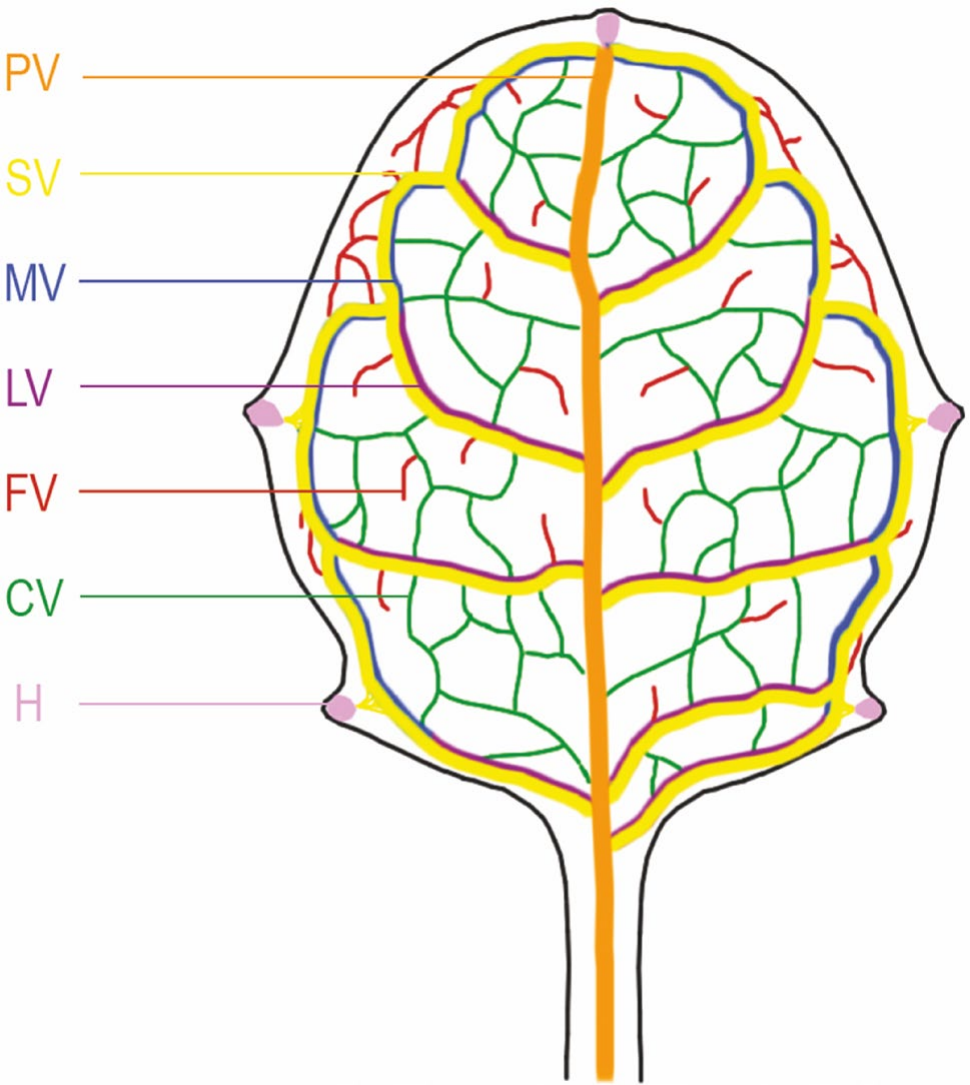
Vascular pattern in first mature Arabidopsis leaf. *PV* primary vein, *SV* loops of secondary veins; *LV* lateral veins, *MV* marginal veins, *CV* tertiary—connected veins, *FV* higher-order—free-ending veins, *H* hydathodes

The vascular system of Arabidopsis leaves is composed of a network of interconnected veins organized in a hierarchical network (Fig. 1; Nelson and Dengler 1997; Candela et al. 1999; Sieburth 1999; Kang and Dengler 2004; Kang et al. 2007). The single primary vein (midvein), which is connected to the vascular system of the stem, extends along the central part of the leaf blade. The secondary veins form closed loops that are connected to the midvein and other secondary veins (Mattsson et al. 1999; Kang and Dengler 2004; Kang et al. 2007). In each of these loops, two regions, which differ in position and formation, can be distinguished. The first region, the so-called marginal vein extends along the margin of the leaf blade, whereas the second region, the lateral vein, runs across the leaf blade and connects the marginal vein to the primary vein (Sawchuk et al. 2008, 2013; Scarpella et al. 2010). The tertiary (connected) veins expand between the existing lower-order veins and usually connect with them at both ends, whereas the higher-order (free-ending) veins are formed as branches of the existing vascular strands and end freely in the mesophyll (Kang and Dengler 2004; Kang et al. 2007; Sawchuk et al. 2008, 2013; Scarpella et al. 2010). The number of vein orders in Arabidopsis can vary in successively initiated leaves (Nelson and Dengler 1997; Scarpella et al. 2004), but the vascular pattern is always repetitive, suggesting that the veins of different orders are genetically programmed. However, several studies indicate that formation of the successive vein orders results from the reiteration of one process common to all vein orders—the branching of procambial strands—and not from the realization of the subsequent stages of the particular genetic program (Scarpella et al. 2004; Marcos and Berleth 2014). It was suggested that the lack of continuation of this mechanism due to the differentiation of mesophyll cells causes the formation of the free-ending highest-order veins (Scarpella et al. 2004).

## Development of the vascular network in Arabidopsis leaves

Formation of the venation network in Arabidopsis leaves is a multistage process that is regulated by auxin and its transport throughout leaf tissues (Mattsson et al. 1999, 2003; Sieburth 1999; Berleth and Mattsson 2000; Aloni et al. 2003; Scarpella et al. 2006; Donner and Scarpella 2009). The cells, predestined to differentiate into conductive elements, are initially morphologically indistinguishable from the other cells of the developing leaf blade, but are genetically determined to the preprocambial identity. Several genes are considered to be markers for the preprocambial stage of vascular differentiation such as *PIN1* (*PIN-FORMED1*), *MP* (*MONOPTEROS*), *ATHB* (*Arabidopsis thaliana HOMEOBOX 8*) and *DOF* (*DNA-BINDING WITH ONE ZINC FINGER*) (Kang and Dengler 2004; Scarpella et al. 2004, 2006; Sawchuk et al. 2007; Wenzel et al. 2007; Donner et al. 2009; Konishi et al. 2015), but their spatio-temporal localization in leaves differs, which may result from their different functions in the preprocambium or/and multistage of this developmental phase (Scarpella et al. 2004; Donner et al. 2009; Konishi et al. 2015).

From morphologically undistinguishable preprocambial cells, continuous elongated strands of narrow procambial cells with dense cytoplasm are formed (Esau 1965; Nelson and Dengler 1997). Two different mechanisms of their early formation have been proposed. According to the first, classical view, which is based mainly on anatomical analyses, the procambium formation is a consequence of coordinated periclinal cell divisions that occur parallel to the axis of emerging veins (Esau 1965; Scarpella et al. 2004, 2006). The second proposed mechanism is based on fluorescent signal analyses in Q0990:mGFP5er enhancer trap line selected for its vein-associated signal considered to be the procambial stage specific (Sawchuk et al. 2007). Because the GFP is detected in already somewhat elongated but not yet longitudinally dividing cells, it was considered that procambial cells acquire a characteristic elongated shape not as a result of divisions, but rather through earlier synchronized cell elongation (Sawchuk et al. 2007; Scarpella et al. 2010). Synchronic expression of the cyclin encoding genes was not observed in the initial strand of procambial cells, which seems to additionally confirm the lack of synchronized longitudinal cell divisions preceding the earliest stage of procambium emergence (Donnelly et al. 1999; Kang and Dengler 2002; Sawchuk et al. 2007).

Variations in the interpretation of the manner of procambial cell formation can result from the use of different definitions of this developmental stage; if we assume that the procambium is a strand of elongated cells with determined growth axes, but with diameters similar to those of surrounding cells, the emergence of the procambium is due to cell elongation; on the other hand, if we consider that the procambium is a strand of elongated and narrow cells, then this cellular phenotype emerges after longitudinal division. Therefore, establishing in detail the spatio-temporal sequence of cellular events (divisions, elongation, differentiation) and the precise definition of all stages of vascular tissue emergence is necessary for a more complete understanding of the mechanisms of venation pattern formation.

**Fig. 2:**
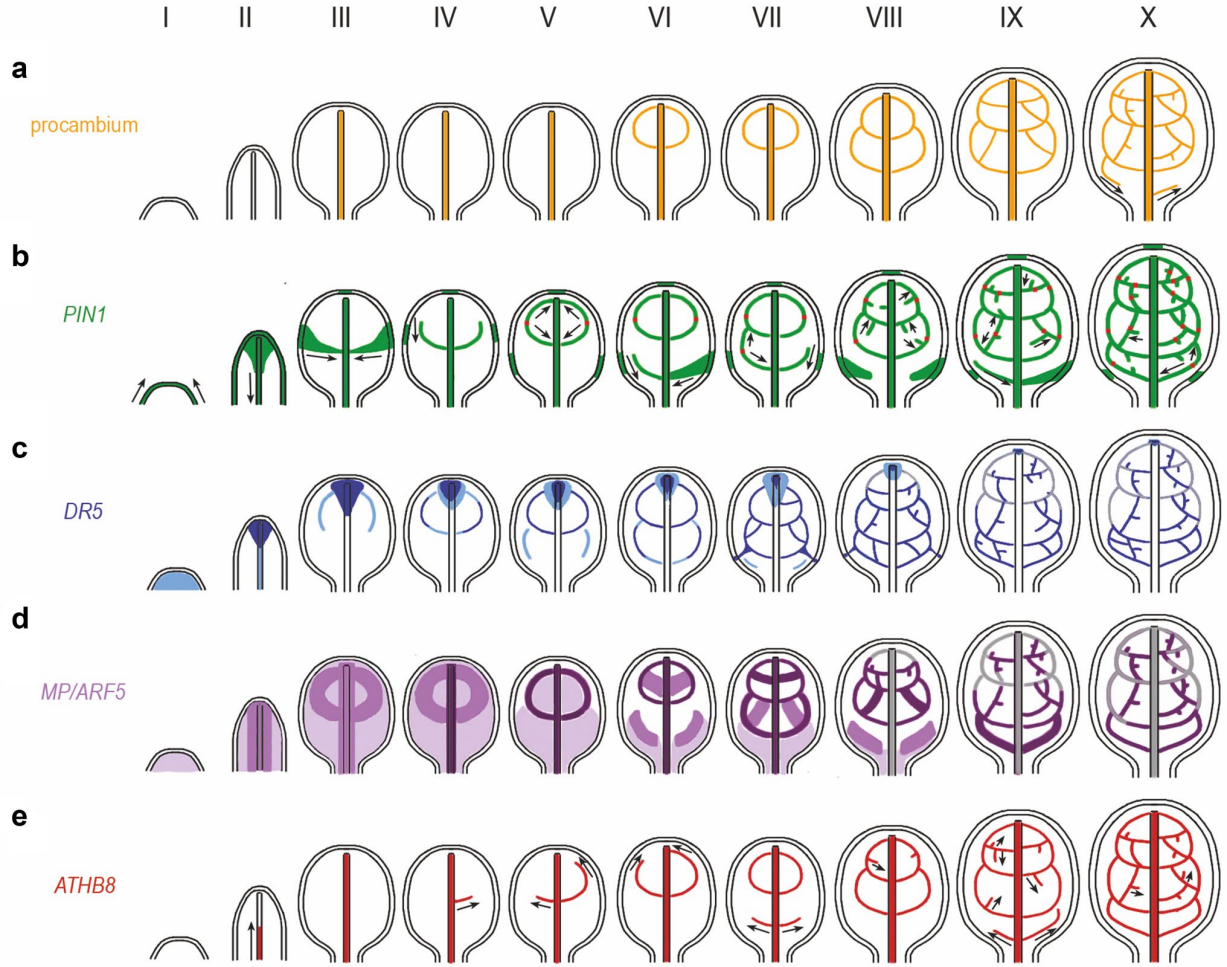
Changes in the pattern of gene expression during procambium development in the first leaf of Arabidopsis. **a** Procambium development; black arrows show the directions of procambium emergence; **b** PIN1; black arrows indicate the direction of PIN1 protein polarity in the plasma membrane; red dots indicate cells with bipolar PIN1 localization; green regions in the epidermis represent auxin convergence points; **c** *DR5* auxin-responsive promoter; the darker blue color shows a stronger *DR5* expression; **d** *MP*; the darker violet color shows a stronger *MP* gene expression; **e** *ATHB8*; black arrows indicate the direction of its progressive appearance; I–X, successive stages of the venation development

The first strand of procambial cells, defined as elongated, but not yet longitudinally dividing cells, emerges in the central part of the leaf blade simultaneously along its entire length, and determines the location of the future primary vein (Fig. 2a III; Scarpella et al. 2004; Sawchuk et al. 2007, 2008). Differentiation of the procambial strands in the first (upper) and second (lower) loops of second-order veins, which starts one day later, is also simultaneous for the whole individual loop (Fig. 2a VI–VIII; Scarpella et al. 2004; Sawchuk et al. 2007). In contrast, the formation of procambial strands in the third loops of secondary veins can proceed either simultaneously along the entire length (Scarpella et al. 2004), or separately in marginal and lateral parts of the loop (Fig. 2a X; Sawchuk et al. 2007). It is the only exception from simultaneous procambial cells development in vascular strands of the first rosette leaf, studied in Arabidopsis, because even in tertiary and higher-order veins, procambium is formed at the same time in entire strands (Fig. 2a IX–X; Scarpella et al. 2004).

It is believed that the manner of procambium formation is related to the level of endogenous auxin. Simultaneous emerging of procambium strands, probably reflects the effective flow of auxin during the initial stages of leaf development, resulting from inefficient biosynthesis of auxin and its low concentrations. On the other hand, nonsynchronous procambium differentiation, separately in marginal and lateral vein of the third loops, can be associated with higher levels of auxin, which is synthesized in adjacent hydathodes (Sawchuk et al. 2007, 2008; Donner et al. 2009; Scarpella et al. 2010). The key role of high auxin concentrations in the nonsynchronous differentiation of the procambium has also been confirmed by experiments involving exogenous applications of auxin and its transport inhibitors (Sawchuk et al. 2007).

Procambial cells forming strands arranged according to a specific spatial pattern subsequently differentiate into functional phloem and xylem elements. Both of these tissues are formed in the same strands and thus have similar arrangements (Carland et al. 1999; Aloni 2001), with the exception of the free-ending veins of the highest-order, which typically lack phloem cells (Aloni 2001).

## The role of auxin transport in leaf vascularization

A considerable body of evidence shows that auxin is involved in all aspects of vascular differentiation (Aloni et al. 2000, 2003; Aloni 2001; De Rybel et al. 2016), including regulation of venation pattern development in leaves (Sieburth 1999; Mattsson et al. 1999; Berleth and Mattsson 2000; Aloni et al. 2003). Vascular tissues differentiate in regions where auxin concentration is elevated (Aloni 2001), and the localization of the intensive auxin flow in the developing leaf reflects the future vascular strand placement (Scarpella et al. 2006; Wenzel et al. 2007; Marcos and Berleth 2014). Moreover, exogenous application of auxin or its transport inhibitors drastically changes venation patterning (Sieburth 1999; Mattsson et al. 1999, 2003; Scarpella et al. 2006).

The essential question, which is yet to be fully resolved, is how auxin regulates the formation of regular patterns of vascular tissue distribution in plants. Two primary theories of the regulatory mechanisms at the transcellular level have been proposed: (1) reaction–diffusion prepattern and (2) canalized auxin flow (Nelson and Dengler 1997; Merks et al. 2007). In both theories, vascular pattern formation is a self-organizing process in which auxin plays a central role (Scarpella et al. 2004). The mechanism of the reaction–diffusion assumes that the pattern of veins is determined locally as a result of interactions between several morphogens, including a long-distance transported inhibitor and a short-distance transported activator (Meinhardt 1976; Merks et al. 2007). This mechanism assumes the possibility of vascular strand fragmentation, leading to the formation of isolated vascular islands. Such phenotypes were identified in few mutants (Koizumi et al. 2000; Steynen and Schultz 2003; Carland and Nelson 2009; Naramoto et al. 2009). The second theory—canalized auxin flow, assumes that the vascular pattern formation occurs along the apical-basal pathways of auxin flow, which is gradually “canalized” into narrow strands. Continuous flow of auxin would reflect the continuity of conductive tissues (Sachs 1969; Merks et al. 2007). The theory of canalization has been confirmed in many mathematical models (Mitchison et al. 1981; Rolland-Lagan and Prusinkiewicz 2005; Merks et al. 2007; Bayer et al. 2009; Lee et al. 2014) as well as in experimental studies (Mattsson et al. 1999, 2003; Sauer et al. 2006; Scarpella et al. 2006; Wenzel et al. 2007). Main support for this theory comes from the characterization of the mechanism driving canalized polar auxin flow (Scarpella et al. 2006; Wenzel et al. 2007; Marcos and Berleth 2014) and the identification of some of its molecular components (Vieten et al. 2007).

Polar auxin transport (PAT) represents auxin movement from cell to cell, and involves the influx and efflux carriers located in the plasma membrane (Zažímalová et al. 2010). Auxin can be transported to the protoplast by diffusion, or by influx carrier proteins from the AUX1/LIKE AUX1 (AUX1/LAX) family (Kerr and Bennett 2007; Adamowski and Friml 2015) while transport from the protoplast to the cell wall is possible only via efflux carriers, mainly from the PIN–FORMED (PIN) family (Friml 2003; Adamowski and Friml 2015). Due to their polar location in the plasma membrane, these proteins determine the direction of auxin flow (Wiśniewska et al. 2006), and their mobility enables dynamic changes in auxin flow direction (Kleine-Vehn et al. 2011). The transport of auxin outside of the protoplast may also be assisted by proteins of the ABC (ATP binding cassette) family, which do not have a polar localization in the plasma membrane, and thus probably do not have a direct regulatory function on the polarity of auxin flow (Mravec et al. 2008; Kaneda et al. 2011).

The auxin transport carriers involved in PAT have tissue and cell-specific localization. Some of them are present in the developing vascular tissues in leaves and appear to be involved in the formation of the venation pattern. Among four of auxin influx carriers from the AUX/LAX family, which are known in Arabidopsis, only one—LAX2 is expressed in leaf at early stages of the vascular tissue differentiation. Interestingly, its expression is related to the procambial stage in veins of all orders with the exception of the primary vein, where LAX2 was never detected (Péret et al. 2012). The function of this protein is not fully understood, but it was suggested that LAX2 proteins contribute to the maintenance of auxin homeostasis essential for normal, symmetrical development of the venation pattern (Moreno-Piovano et al. 2017).

The import role for the vascular pattern development seems to have the auxin carriers from the PIN family. Eight proteins of the PIN family have been identified in Arabidopsis. These proteins differ in the length of the hydrophilic loop inside the polypeptide chain and in the cellular localization (Zažímalová et al. 2010; Ganguly et al. 2014). The PIN1-PIN4 and PIN7 proteins, which have a long hydrophilic loop, are located in the cell membrane and determine the direction of the intercellular auxin flow (Vieten et al. 2007; Zažímalová et al. 2010), whereas PIN5, PIN6, and PIN8 proteins with shorter hydrophilic loops are located in the membrane of the endoplasmic reticulum (ER) and may play a role in regulating auxin levels in the cytosol (Mravec et al. 2009; Sawchuk and Scarpella 2013a). Both groups of PIN proteins seem to be involved in the regulation of the venation pattern development in leaf.

### The role of plasma membrane associated PIN proteins in leaf vascularization

Four of the plasma membrane associated PIN proteins (PIN1, PIN3, PIN4, and PIN7) are expressed in the first leaf of Arabidopsis and are able to potentially regulate the direction of auxin transport during vascularization (Scarpella et al. 2006). Only PIN1 is active in the earliest stages of venation development (when vein arrangement is determined), and mutations of *PIN1* gene result in drastically modified vascular pattern (Mattsson et al. 1999). Other PIN proteins identified in leaves and associated with the cell membrane, are expressed only following the completion of procambium formation, and therefore, most likely do not have a role in the process of venation pattern formation (Scarpella et al. 2006). For this reason, PIN1 proteins are considered the most important for polar auxin transport regulating the development of vascular patterns in leaves.

Expression of the *PIN1* gene appears in the earliest stages of vascular tissue formation and is considered to be a marker for preprocambial cells (Scarpella et al. 2006; Scheres and Xu 2006). Spatio-temporal analyses of the location of PIN1 proteins throughout the entire leaf development facilitated the determination of main sources and flow directions of auxin at all stages of the venation formation, and led to a better understanding of the mechanisms regulating this process. PIN1 proteins are expressed in the L1 layer of shoot apical meristems (SAM) at the site of future leaf primordia, before the morphological signs of organogenesis are visible (Reinhardt et al. 2003). During the earliest stage of leaf primordium emergence, PIN1 proteins occur in all surface-layer cells with acropetal polarity (Fig. 2b I), and are responsible for auxin transport to the top of the primordium (Reinhardt et al. 2003), where the first “convergence point of auxin” (site of auxin accumulation by its depletion from surrounding cells) is formed (Scarpella et al. 2006, 2010; Wenzel et al. 2007) that will over time convert into the hydathode at the leaf tip. Later, PIN1 expression appears inside the primordium, in the region of the future primary vein, forming a domain wider at the top of the leaf and narrower at the base, with the polarity clearly basal and toward the already existing leaf trace (Fig. 2b II; Reinhardt et al. 2003; Scarpella et al. 2006; Wenzel et al. 2007). In the next stage of leaf development, consecutive convergence points of auxin that supply the developing secondary veins with this hormone are formed basipetally in the epidermis of the leaf blade edges (Fig. 2b III-X; Scarpella et al. 2006, 2010; Wenzel et al. 2007; Marcos and Berleth 2014). The convergence points for the first and usually also the second pair of loops are transient, but for subsequent loops these points turn into hydathodes. The PIN1 expression domains of secondary vein loops are formed in two stages: in the first, the domain connecting the convergence point on the leaf margin with the primary vein domain is formed, which corresponds to the localization of the future lateral vein (Fig. 2b III, VI, VIII, IX; Scarpella et al. 2006). This domain is broad near the convergence point and narrows toward the midvein, in accordance with PIN1 polarity. Over time, this entire domain becomes narrow and its connection with the convergence point disappears (Fig. 2b III–X; Scarpella et al. 2006). In the second stage, the PIN1 expression domain corresponding with the future marginal vein develops, emerging from the existing lateral PIN1 domain and extending acropetally toward the leaf tip, where it connects to the primary vein domain or, if present, the secondary vein domain (Fig. 2b IV–X). The polarity of the PIN1 proteins in the marginal region is at first basipetal, toward the lateral domain, and it partially changes when the PIN1 domain of the whole secondary loop is completed. As a consequence, the marginal domain consists of two fragments with opposing PIN1 polarity, which are connected by a single bipolar cell (Fig. 2b V–X; Scarpella et al. 2006). The PIN1 domains of higher-order veins extend progressively from the pre-existing domains (Fig. 2b VIII–X; Scarpella et al. 2006; Wenzel et al. 2007; Marcos and Berleth 2014). In the veins freely ended in the mesophyll, the PIN1 expression domains develop from pre-existing strands and also end freely, with the PIN1 polarity always basal in relation to the pre-existing strands (Fig. 2b VIII–X); in the connecting veins, the PIN1 expression domains extend from two different pre-existing strands and link with each other. Thus, one domain is formed that consists of two fragments with opposing PIN1 polarity that are connected by a single bipolar cell, as in the secondary vein loops (Fig. 2b IX–X; Scarpella et al. 2006; Marcos and Berleth 2014). The new PIN1 expression domain can also branch from one preexisting PIN1 expression domain and extends sometimes contacting with other pre-existing PIN1 expression domain (Marcos and Berleth 2014).

Changes in the PIN1 localization pattern are evidence that the polar auxin transport involving these proteins determines the site of vascular tissue formation during leaf development. The expression of PIN1 in the form of broad domains gradually narrowing to thin strands confirms the canalization hypothesis (Scarpella et al. 2006; Wenzel et al. 2007; Linh et al. 2018). Additional support for this hypothesis is provided by the PIN1 expression domains in the *van3* (*vascular network defective3*) mutant (Scarpella et al. 2006), which was at first considered as an evidence for the diffusion–reaction mechanism (Koizumi et al. 2000). In the very early stages of vascular development in this mutant, the PIN1 expression domains form a continuous pattern consistent with normal vein arrangement. Fragmentation of these domains, and therefore, also the differentiated vascular tissues occurs later in leaf development, after the main vascular pattern is established (Scarpella et al. 2006; Naramoto et al. 2009).

Polarization of PIN1 proteins that transport auxin along the future veins is always directed toward the pre-existing vascular strand (Sawchuk and Scarpella 2013b; Scarpella 2017); thus, the existing vascular system probably plays an important role in the direction of auxin transport. It is generally believed that the existing vasculature drains auxin from its sources, such as convergence points and growing regions of the leaf blade, and that this process is essential for the formation of continuous vascular strands (Scarpella et al. 2006; Bayer et al. 2009; Linh et al. 2018). Notably, the spatio-temporal changes in PIN1 polarization relating to pre-existing vascular strands suggest that these strands acquire the ability to drain auxin during the procambial stage of development (compare, for example, Fig. 2a III with Fig. 2b III, and Fig. 2a VI with Fig. 2b VI). This is interesting from the perspective of two proposed mechanisms for the polarization of PIN1 proteins (Bayer et al. 2009; Feller et al. 2015). The first mechanism, “up the gradient”, assumes that polarization of PIN1 proteins occurs toward the highest auxin concentration, and it was suggested for the organogenesis at SAM and the formation of convergence points during leaf development (Reinhardt et al. 2003; Scarpella et al. 2006; Heisler et al. 2005; Jönsson et al. 2006; Smith et al. 2006; Bayer et al. 2009; Marcos and Berleth 2014). The second mechanism, “with the flux”, assumes polarization of PIN1 proteins in accordance with the direction of auxin transport but toward low auxin concentrations, and it was proposed for the continuous vascular pattern formation (Sachs 1969; Mitchison et al. 1981; Bayer et al. 2009; Feller et al. 2015). It is likely that two primarily separated processes, organogenesis, and vascularization, became regulated in Arabidopsis by the same PIN1 proteins due to evolutionary loss of one from two functionally distinct PIN1 clades, which probably occur in the most angiosperms. Such a functional fusion could explain the putative different PIN1 polarization mechanisms in the regulation of various developmental processes (O’Connor et al. 2017). However, some studies have raised questions about the existence of two different, opposite mechanisms for the polarization of PIN1 proteins (Merks et al. 2007; Stoma et al. 2008). Many experimental data suggest that new vascular strands are initiated by high auxin level (Mattsson et al. 2003; Bayer et al. 2009), indicated by the expression of synthetic auxin-responsive promoter *DR5* and *PIN1* or *ATHB8* genes, which are present in these strands. Their expression is maintained after procambium formation with the exception of *DR5* in the midvein, suggesting that the procambial cells have a relatively high auxin level in relation to the surrounding tissues. Taking this into consideration, the formation of PIN1 expression domains with the polarity directed toward existing procambial strands indicates that PIN1 proteins can be polarized toward cells with high auxin level during the vein development. This suggests the common mechanism of PIN1 proteins polarization for both processes, organogenesis and vascular pattern formation, always toward the high auxin concentration.

Our knowledge on different levels of intracellular regulation of PIN1-dependent auxin transport, which affect leaf vascularization, has recently increased. Among others, it has been shown that the AtMPK10 kinase, and its upstream kinase AtMKK2, are involved in the regulation of vascular pattern complexity, probably by affecting vesicle trafficking of PIN1 to the cell membrane, and thereby having an impact on PAT efficiency (Stanko et al. 2014). In other study, *UNHINGED* (*UNH*) gene encoding the Golgi-associated retrograde protein (GARP) complex has been identified as also involved in venation development (Pahari et al. 2014). The *unh-1* mutant has extensive PIN1 expression domains in auxin convergence points and the altered pattern of leaf vascularization. It has been suggested that UNH regulates PIN1 protein levels in marginal cells of the leaf blade through the targeting of PIN1 for degradation in the lytic vacuole. These studies indicate that degradation of PIN proteins can be another important level of regulation of auxin flow, which is responsible for the development of vascular tissues (Pahari et al. 2014).

### The role of ER-associated PIN proteins in leaf vascularization

Recent studies have shown that ER-associated PIN proteins: PIN5, PIN6 and PIN8 can also play an important role in the regulation of leaf vascular pattern development (Sawchuk et al. 2013; Sawchuk and Scarpella 2013a; Verna et al. 2015). All of these proteins are present in the developing leaf and their localization is strongly associated with successively emerging veins. Expression of PIN6 appears the earliest, overlapping in the subepidermal layers with PIN1 expression domains, while the expressions of PIN8 and PIN5 are detectable 2–3 days later, respectively, and are limited to single rows of vascular cells (Sawchuk et al. 2013; Verna et al. 2015). Interestingly, the expression domains of PIN5, PIN6, and PIN8 are not related to the same cells; they are mutually exclusive, but are all located within the wide domain of PIN1 expression (Verna et al. 2015). Based on these findings it has been proposed that venation pattern development is controlled by two distinct, convergent auxin transport pathways: (1) intercellular polar auxin transport mediated by the plasma membrane-located PIN1 proteins; and (2) intracellular auxin transport involving ER-associated PIN5, PIN6, and PIN8 proteins (Sawchuk et al. 2013). Auxin transport by ER-associated PIN proteins probably has a regulatory function in the determination of vein density (Verna et al. 2015). The PIN6 and PIN8 proteins decrease PIN1-dependent vein formation, and have a redundant function in this process, while PIN5 promotes the vascular strand formation, acting antagonistically to PIN6 and PIN8. All of these proteins perform their functions in the regulation of vein density probably by controlling the intracellular auxin levels available for the receptors, and thereby determining PIN1 protein abundance and PIN1-dependent intensity of the auxin drainage, which are necessary for vein formation (Verna et al. 2015). The PIN6 and PIN8 proteins, similar to PIN1, also appear to have a function in the process of connecting veins into the network, although the way they are involved in this process is not clear (Verna et al. 2015). A complete understanding of the role of ER-associated PIN proteins in leaf vascularization requires further research.

## The role of auxin signaling in leaf vascularization

Many genes have been identified that appear to be involved in various processes associated with the formation of functional vascular tissues, including genes that are active when the vascular pattern is determined (Caño-Delgado et al. 2010; Gandotra et al. 2013). Most of these are regulated by auxin (Baima et al. 1995; Mattsson et al. 2003) in the signaling pathway associated with the TRANSPORT INHIBITOR RESPONSE/AUXIN SIGNALING F-BOX (TIR/AFB) auxin receptors (Donner et al. 2009), which are part of the ubiquitin ligase complex SCF^TIR/AFB^ (Guilfoyle 2015). At high concentration, auxin binds to the receptor and leads to the degradation of the repressor proteins from the Aux/IAA family that formed dimers with auxin response factors (ARFs), inhibiting their activity. ARFs released in this way bind to unique sequences, auxin response elements (AuxRE) in promoters of auxin response genes, where they function as activators or repressors of these genes transcription (Salehin et al. 2015; Trenner et al. 2017). Recently, several additional ARF-binding sites have been identified in the gene promoters, which can also be involved in the process of auxin signaling via the TIR/AFB receptor (Zemlyanskaya et al. 2016; Weijers and Wagner 2016).

### Detection of auxin signaling during leaf vascularization

The TIR/AFB-dependent mechanism of auxin signaling was used in the engineering of auxin response biosensors. One of these is the synthetic auxin-induced promoter *DR5*, which contains several AuxRE binding ARFs in response to increase in the auxin concentration and degradation of Aux/IAA repressor proteins (Ulmasov et al. 1997; Schenck et al. 2010). *DR5* fused to reporter genes is a widely used marker for the monitoring of auxin response in plant cells (Chapman and Estelle 2009). The use of transgenic lines with *DR5* was of great importance for better understanding auxin-regulated developmental processes in plants. Interestingly, recent studies indicated the presence of regulatory sequences other than AuxRE that can induce auxin responses many times stronger than does AuxRE, which are present in *DR5* promoter (Liao et al. 2015; Weijers and Wagner 2016). Thus, *DR5* expression does not necessarily reflect the full cellular response to auxin. Despite this, DR5 is still a very useful tool in the study of auxin-dependent processes.

Transgenic lines containing *DR5* promoter fused to different reporter genes were also used to analyze changes in the localization of the auxin response during successive stages of leaf development (Fig. 2c; Mattsson et al. 2003; Steynen and Schultz 2003; Hou et al. 2010; Marcos and Berleth 2014). In the young leaf primordium, *DR5* expression is weak throughout the entire emerging leaf, excluding the epidermis (Fig. 2c I); later, expression of *DR5* reporter becomes the strongest in the subepidermal cells at the top of the developing leaf and weaker towards the base of the primordium, thereby defining the region of the future primary vein (Fig. 2c II; Mattsson et al. 2003; Steynen and Schultz 2003). Together with the growth of the primordium, expression in the primary vein disappears from the base but remains high at the top of the leaf blade (Fig. 2c III). At the same time, two additional *DR5* expression domains emerge parallel to the leaf margin and connect with the strong *DR5* response site at the leaf top, determining the marginal fragments of the first pair of secondary vein loops (Fig. 2c III; Mattsson et al. 2003; Hou et al. 2010). The *DR5* expression domains for the lateral veins of these loops develop through connections of the terminal regions of the marginal domains with the midvein via groups of cells expressing the *DR5* (Fig. 2c III, IV; Mattsson et al. 2003). Initially, the domains of entire loops are characterized by heterogeneous intensity of the expression signal, which increases and becomes more uniform as the procambial strands mature (Fig. 2c III–V; Mattsson et al. 2003). Similar changes in the pattern of *DR5* expression precede the formation of each subsequent, basipetally added loops of secondary veins (Fig. 2c V–VIII; Mattsson et al. 2003; Steynen and Schultz 2003). Domains of *DR5* response also emerge in the areas between the lower-order veins, determining the regions for future procambial strands of tertiary and quaternary veins (Fig. 2c VII–X; Mattsson et al. 2003; Steynen and Schultz 2003). Expression of the synthetic auxin responsive reporter *DR5* always precedes the stage of procambial strands formation and stops as vascular tissues are matured (Mattsson et al. 2003), indicating that differentiation of vascular tissues occurs in regions with enhanced auxin concentrations, a necessary factor for this process.

### The role of the *MP* gene in leaf vascularization

The *MP* gene is a member of the ARF gene family and is also known as *ARF5* (Hardtke et al. 2004). It is considered to be a key regulator of leaf vascularization (Berleth and Jürgens 1993; Przemeck et al. 1996; Mattsson et al. 2003; Wenzel et al. 2007) because its activity is induced by auxin (Mattsson et al. 2003; Donner et al. 2009), its transcript distribution pattern in leaves is related to the developing vascular system (Wenzel et al. 2007), and mutation in this gene causes, in addition to numerous other developmental defects, drastic reduction of the venation network (Berleth and Jürgens 1993; Przemeck et al. 1996; Mattsson et al. 2003).

Expression of the *MP* gene, if refer to the *MP* mRNA transcript level, is detectable in the internal layers of the organogenic zone of the SAM, in the region of the incipient primordium prior to its emergence (Wenzel et al. 2007). In young developing primordium, the *MP* expression domain encompasses all subepidermal cells (Fig. 2d I; Hardtke and Berleth 1998; Hardtke et al. 2004; Wenzel et al. 2007). Later, the level of expression of this gene increases in the wide central region of the primordium, within which the narrow primary vein will be form (Fig. 2d II; Wenzel et al. 2007), which was confirmed on the level of the MPpro:MP:CFP fusion protein localization (Donner et al. 2009; Krogan et al. 2012). Then, two broad *MP* transcription domains appear between the central domain and the margin of the leaf blade, on both sides simultaneously, defining the marginal regions of the first pair of the secondary vein loops (Fig. 2d III; Wenzel et al. 2007). The broad domains related to the midvein and the secondary veins are gradually restricted to thin strands, within which the *MP* mRNA level increases (Fig. 2d II–V; Wenzel et al. 2007). The same sequence of events occurs in the subsequent secondary loops that are added basipetally (Fig. 2d VI–X). With respect to higher-order veins, *MP* expression initially forms large domains between the domains of the primary and secondary veins and then narrows down to the width of the preprocambial strands (Fig. 2d VI–X; Wenzel et al. 2007). In all domains, *MP* transcription is the highest in isodiametric preprocambial cells, slightly lower in elongated procambial cells, and the weakest or completely absent in fully differentiated vascular elements (Wenzel et al. 2007).

Expression of the *MP* gene during venation development always initially appears in the form of broad domains, within which the narrow domains of auxin flow and auxin response occur, as indicated by the expression patterns of PIN1 and auxin responsive reporter *DR5* (Fig. 2b–d). Later expression of *MP* is restricted to these narrow strands and its level increases, suggesting that *MP* is induced by the auxin flowing in that region.

### The role of the *ATHB8* gene in leaf vascularization

The *ATHB8* gene is a member of the HD-ZIP III gene family. Genes from this family encode five transcription factors: REVOLUTA (REV), PHABULOSA (PHB), PHAVOLUTA (PHV), CORONA (CNA), and ATHB8, which bind to DNA, regulating of the gene expression in many developmental processes (Baima et al. 2001; Prigge et al. 2005). During vascularization, they are involved in vascular tissue specification, vascular pattern formation, cell division and cell-differentiation, and at least in some of these processes can have a redundant function (Prigge et al. 2005; Ramachandran et al. 2017).

Expression of the *ATHB8* gene is induced by auxin and in leaves is associated with the early stages of vascular tissue development, that is, development of the preprocambium and procambium. It is assumed that *ATHB8* has different functions during each of these stages (Baima et al. 2001; Donner et al. 2010; Ramachandran et al. 2017). During the preprocambial stage, its activity probably relates to vascular pattern formation, whereas during the procambial stage, it may be involved in the regulation of meristematic cell divisions and early xylem specification, acting simultaneously as a negative regulator of xylem differentiation (Baima et al. 2001; Scarpella et al. 2004; Prigge et al. 2005; Donner et al. 2009; Ramachandran et al. 2017).

During the early stages of leaf development, *ATHB8* gene expression appears acropetally in the non-differentiated cells, in the region of the future primary vein (Fig. 2e I–III; Scarpella et al. 2004). The *ATHB8* expression domains, which correspond to the first pair of the secondary vein loops, become visible at approximately 1 day later (Fig. 2e IV–VI). These domains extend progressively from the middle part of the central vein, first toward the leaf margins determining the domains of the lateral veins, and later acropetally along the margins indicating the domains of the future marginal veins. The extending domains close the loops connecting with the midvein at the top of the leaf (Fig. 2e VI, VII; Scarpella et al. 2004). The *ATHB8* expression domains of subsequent secondary loops always appear in the basal sequence and always occur progressively, from the central vein to the margin of the lamina and then acropetally, connecting to the expression domains of the earlier loops (Fig. 2e VII–X). In the regions between the existing lower-order veins, *ATHB8* expression domains develop, which define the regions of the tertiary-connecting veins and the quaternary-free-ending vein regions, which diverge progressively from existing vascular strands (Fig. 2e VIII–IX; Scarpella et al. 2004).

Expression of the *ATHB8* gene is always progressive and extends from pre-existing veins (Scarpella et al. 2004; Donner et al. 2009). Interestingly, this expression always appears in the direction opposite to that of PIN1 protein polarity and thus to the direction of auxin flow, which is difficult to explain, since the *ATHB8* gene is induced by auxin. Moreover, there is no clear correlation between changes in the *DR5* expression pattern, indicating a high level of auxin and the auxin response, and the progressive extension of the *ATHB8* expression domains (Fig. 2c, e), nor is there correlation between *DR5* expression patterns and other transcription factors from the HD-ZIP III family (Ramachandran et al. 2017). As such, it has been suggested that some factors other than auxin may participate in the activation of *ATHB8* and other genes of the HD-ZIP III family (Ramachandran et al. 2017). The lack of a clear correlation between the *DR5* and *ATHB8* expression patterns may also be because the AuxRE present in the *DR5* synthetic promoter and the ARF-binding sequence in *ATHB8* are not identical (Donner et al. 2009). Recently it has been shown that different sequences in the auxin-sensitive gene promoters can induce varied auxin responses (Liao et al. 2015; Weijers and Wagner 2016).

## Regulation of leaf vascular pattern development

The factors considered to be involved in the formation of the venation pattern in the leaves, are auxin, its polar transport (in which the PIN1 proteins are involved) and the *MP* and *ATHB8* genes, which are induced by auxin (Mattsson et al. 2003; Scarpella et al. 2006; Wenzel et al. 2007; Donner et al. 2009). Recent studies have provided additional information on the possible interactions between all of these factors, but their exact role in the vascularization process has yet to be fully elucidated.

It is suggested that the *MP* gene regulates expression of *PIN1*, because the expression domains of these genes partially overlap during leaf vascularization, and the mutation in the *MP* gene reduces the levels of PIN1 proteins (Wenzel et al. 2007). Based on these results, a positive feedback mechanism has been proposed between auxin flow, PIN1 expression and polarization, and *MP* activity (Scarpella et al. 2006; Wenzel et al. 2007; Campbell and Turner 2017). Accordingly, the positive feedback loop is initiated by increasing auxin concentration in the cells. The auxin produced in the epidermis and subepidermal cells of growing leaf regions initially induces the broad region of low PIN1 expression with their nonpolar localization, which partially overlaps with the broad domain of the relatively weak *MP* expression. During the canalization of auxin flow to narrow strands, expression of the *MP* gene increases, which enhances expression of PIN1 proteins and their polarization. In response, auxin transport increases, inducing even stronger expression of *MP*. Direct interaction between MP and *PIN1* has recently been shown to occur during organogenesis. MP can induce *PIN1* by binding to a sequence in its promoter, which is almost identical to the AuxRE (Krogan et al. 2016). The cells of the leaf blade, defined as preprocambial due to *PIN1* gene expression, may still lose preprocambial identity and differentiate into other types of cells. It is, therefore, believed that the MP-induced expression of *PIN1* determines the differentiation of cells in vascular tissues in a way that can be reversible. This, in turn, allows for corrections to be made during development of the vascular pattern in the event of disturbance to auxin transport (Donner et al. 2009). Interestingly, *MP* expression during leaf development is always associated with central tissues and has never been observed in epidermal cells, despite the presence of auxin and PIN1 expression (Fig. 2b, d; Wenzel et al. 2007), suggesting that PIN1 in the epidermis can be induced in a different, MP-independent way.

In the region of the broad domains of *MP* and *PIN1* expression, the narrow domains within which the *ATHB8* gene is active appear (Donner et al. 2009, 2010). Based on changes in the pattern of *ATHB8* expression during leaf development it was suggested that ATHB8 irreversibly determines the morphologically indistinguishable, isodiametric preprocambial cells to differentiate into the procambial cells (Scarpella et al. 2004; Donner et al. 2009; Gardiner et al. 2011). However, it was also proposed that *ATHB8* can facilitate rather than ultimately determine the differentiation into procambium (Marcos and Berleth 2014). The function of the *ATHB8* gene in the process of leaf vascularization has not yet been precisely defined.

Evidences suggest that *MP* induces not only *PIN1* but also *ATHB8* gene expressions (Mattsson et al. 2003; Donner et al. 2009, 2010; Campbell and Turner 2017). It has been shown, for example, that the *MP* gene can bind directly to the *ATHB8* promoter in the region of the TGTCTG sequence, which is very similar to the AuxRE (Donner et al. 2009, 2010). However, the association of this interaction with the *ATHB8* gene function is not fully understood. Mutation of the *ATHB8* gene in the *mp* mutant enhances the phenotypic effect of the vein pattern disorder that normally occurs in this mutant, whereas mutation of the *ATHB8* gene alone does not alter venation patterning and expression of the *MP* gene (Donner et al. 2009). It is believed that the effect of the lack of the *ATHB8* gene function may be masked by *MP* activity, which may also activate other genes from the HD-ZIP III family (Donner et al. 2009, 2010).

One important question is why the *ATHB8* gene is activated only in the narrow domain of future procambial cells and not in the broader domain of *MP* gene expression if this gene is its direct activator (Donner et al. 2010). Three possible reasons for this have been proposed: (1) MP may require an additional activator that is present only in cells that are determined to the procambial identity; (2) in the determinate procambial cells, some unknown inhibitor of *ATHB8* expression can be removed (3) MP proteins may occur at different concentrations in different regions of the leaf primordium and only the future procambial cells have these proteins in concentrations sufficient to activate *ATHB8* gene expression (Donner et al. 2010). Interactions between the *ATHB8* and *MP* genes may be even more complicated. Both genes belong to large gene families, and the other genes from these families can act redundantly (Campbell and Turner 2017) and may also play an important role in venation development. In addition, MP proteins have been shown to activate the *ATHB8* gene during leaf vascularization (Donner et al. 2009), but different interactions have been shown to occur during the process of vascular patterning and xylem differentiation in roots, in which *MP* was induced by PHABULOSA (PHB) proteins from the same family as ATHB8, which can bind to its promoter (Müller et al. 2016). Interactions between members of these two ARF and HD-ZIP III transcription factor families can, therefore, be bidirectional and changeable over the course of leaf venation pattern development.

It has recently been demonstrated that during the earliest stages of venation development, MP can also induce the expression of the *AtDof5.8* gene (Konishi et al. 2015), which is a member of the family of genes encoding the zinc finger transcription factors (Le Hir and Bellini 2013). Expression of this gene is induced by auxin already in the preprocambial stage, when MP binds to the AuxRE sequences in its promoter (Konishi et al. 2015). It is presumed that the *AtDof5.8* gene is another important element of the mechanism of leaf vascularization, with a very complex function that has not yet been deciphered but the understanding of which may help in the future elucidation of that process (Konishi et al. 2015; Konishi and Yanagisawa 2015).

Polar transport of auxin is undoubtedly very important for the formation of vascular tissues, but it is also important that it is interconnected with many other factors involved in this process. Studies with simulations of vascular development in growing leaves have shown that the canalized auxin flow itself is not sufficient for proper formation of the venation pattern. Appropriate modifications to cell divisions and the elasticity of cell walls that are likely to be dependent on auxin flow or cell-differentiation status are probably also necessary for proper vascular pattern formation (Lee et al. 2014). The importance of the coordination of venation development and leaf tissue growth has also been demonstrated in transgenic Arabidopsis lines with ectopic cell divisions in ground and vascular tissues (Wenzel et al. 2012). In both cases, ectopic divisions resulted in severe changes in the venation pattern.

Another important and relatively unknown component of the regulation of venation development is the process of auxin biosynthesis (Aloni et al. 2003; Cheng et al. 2006). Recently, a map of spatio-temporal changes in the expression of genes responsible for auxin biosynthesis on the IPA (indole-3-pyruvic-acid) pathway has been presented for the leaf (Kneuper et al., unpublished). It has been found that vein initiation is closely correlated with the expression domains of the enzymes encoded by these genes. A model was then constructed that tested the importance of auxin biosynthesis, auxin transport, and mechanical factors in the vein formation process. Consequently, it was proposed that local auxin biosynthesis in emerging veins is an integral part of the mechanism that regulates the development of vascular pattern in the leaf (Kneuper et al., unpublished).

Interestingly, recent studies using the chemical biology approach to identify the components of developmental signaling pathways allowed recognizing several new active molecules that alter the leaf venation pattern in different ways. Their actions directly or indirectly influence the auxin-dependent regulation of this process, but at the same time indicate the presence of as-yet unidentified regulators of vascular network formation (Carland et al. 2016).

## Summary and perspectives

**Fig. 3:**
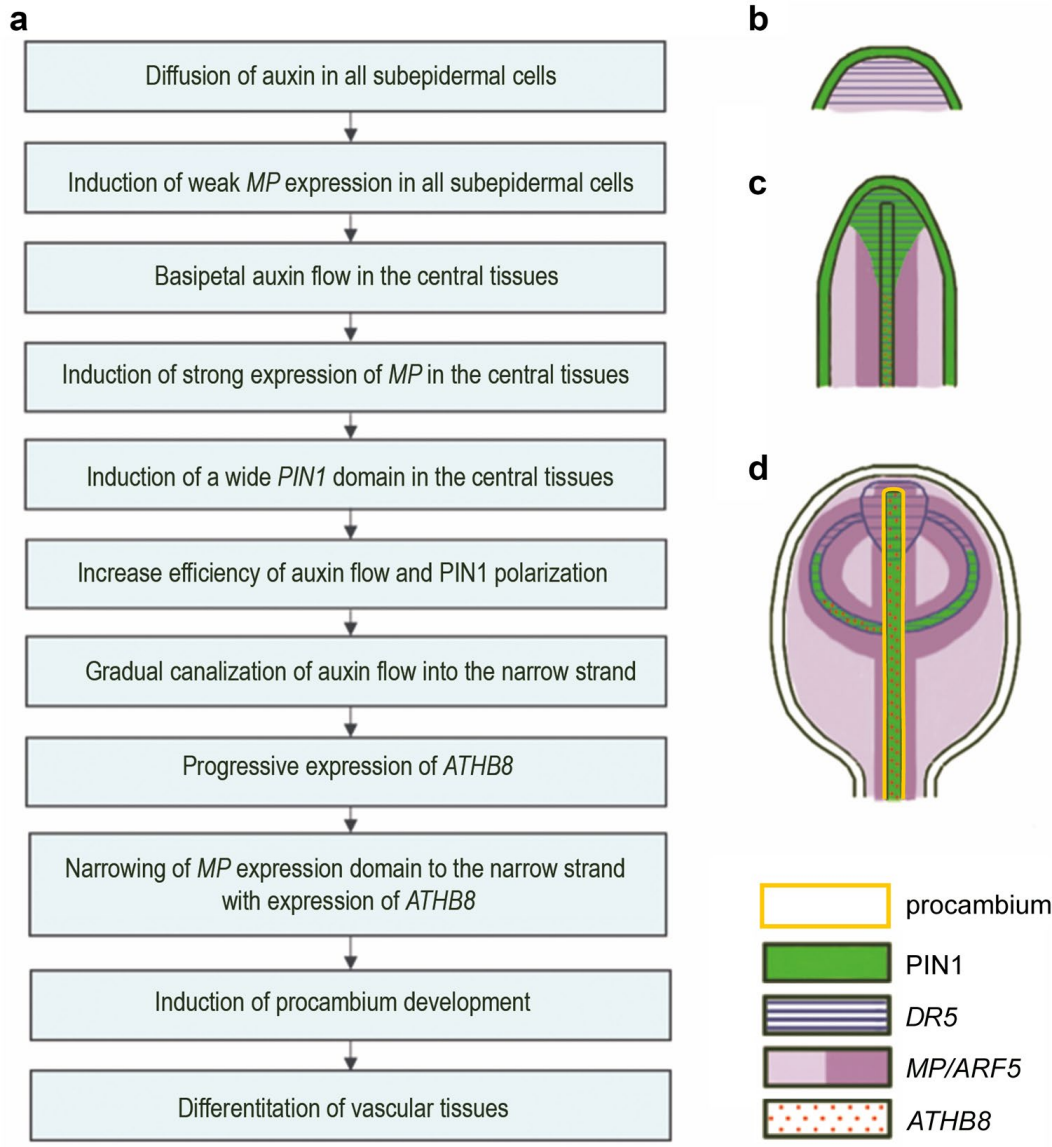
Regulation of the early stages of leaf vascularization in Arabidopsis. **a** Sequence of events during the formation of the central vein. **b**–**d** Merge image of procambium and expression patterns of genes involved in vascular differentiation in the successive stages of leaf development

In this paper, we present published experimental data concerning changes in gene expression patterns during the early stages of leaf vascularization. This allowed us to propose the sequence of events leading to vascular pattern development in leaves (Fig. 3a). For clarity, we describe these events solely in the context of primary vein development, but the sequence appears to be similar for veins of all orders.

### Sequence of events during leaf vascularization

- Auxin in low concentrations diffuses in the subepidermal layers of the developing primordium and induces weak expression of the *MP* gene. Low levels of auxin are indicated by weak signal of the auxin responsive reporter *DR5::GUS*. At the same time, the auxin is transported by PIN1 proteins acropetally in the epidermis to the top of the developing leaf, but in this tissue does not induce expression neither of *MP* nor *DR5* (Fig. 3b).
- Auxin accumulating at the top of the primordium enters subepidermal cells and flows basipetally in the broad central region to the base of the leaf, thereby determining the region of the future primary vein. Most likely this is partially due to polar transport involving PIN1 proteins and partially due to diffusion (Fig. 3c).
- Additional auxin in the broad central domain induces stronger expression of the *MP* and *DR5*. Notably, the expression domains of both of these genes overlap only in the top region of the leaf blade, as in the basal direction, the *MP* expression domain is much wider than that of *DR5* (Fig. 3c). Possibly these genes differ in their sensitivity to auxin.
- Auxin that flows in the central region of the leaf blade also induces the expression and polar localization of PIN1, thereby regulating their own transport and its canalization to the narrow strand, which determines the region of the primary vein. It has also been proposed that *PIN1* expression is induced by the MP transcription factor by the positive feedback mechanism. However, the expression domains of *MP* and *PIN1* at this stage of development overlap only in the top region of the leaf, whereas the expression domain of *PIN1* is narrower than that of *MP* in the basal direction (Fig. 3c), suggesting that MP induces *PIN1* only in the presence of polarized auxin flow.
- Polarized auxin transport in the central tissues of the leaf blade induces *ATHB8* gene expression. Transport of auxin is gradually channeled into the narrow strand and directed toward the existing vasculature, which means that the narrow domain with the clear PIN1 polarity firstly occurs in contact with existing vascular tissues, and is then formed toward the apical region of leaf (Fig. 3d). The progressive expression of *ATHB8* is always consistent with this direction and always opposite to the direction of PIN1 polarity; it can be assumed, therefore, that the only narrow *PIN1* expression domain and polarization of this protein may be involved in inducing *ATHB8* gene expression. Previous studies have demonstrated direct induction of preprocambial *ATHB8* expression by MP transcription factors, but why the broad domain of *MP* expression induces only the narrow strand of the *ATHB8* activity is unknown. Localization of *MP* expression in the developing leaves was carried out mainly by in situ hybridization, a method that fails to reveal whether MP is the active transcription factor throughout its expression domain. It is likely that within this entire domain only the narrow strand has active MP released from the Aux/IAA repressor in response to the high concentrations of auxin transported within this strand (Ckurshumova et al. 2012). As such, MP proteins induce the expression of the *ATHB8* only within this narrow region, which determines the preprocambium strand of the future primary vein.
- Narrowing of the *MP* expression domain in the central part of leaf, and as a result its strong co-expression with *ATHB8*, precedes the emergence of the procambium of the primary vein; thus, it is possible that their interaction regulates procambium development.

In the sequence of events outlined here, auxin and its polar transport play primary and central roles. However, despite substantial evidence about the association of auxin transport via polarized PIN1 proteins with the formation of vascular tissues, the mechanism of PAT-dependent vascularization is still not fully elucidated and remains very important goal for future research. Moreover, some studies have shown that vascular differentiation may occur without the involvement of PAT and in the absence of PIN1 proteins in plasma membranes, which strongly suggests the existence of additional mechanisms regulating vascular development (Banasiak 2011; Kierzkowski et al. 2013). It is highly possible that such an important process as vascular system differentiation is regulated by more than one mechanism, a view supported by the rarity of discontinuities in vascular tissues that would prevent their functioning. Even in mutants with such discontinuities as *mp, cvp* (*cotyledon vein pattern*), *fkd* (*forked*), *van3*/*sfc* (*scarface*) (Hardtke and Berleth 1998; Steynen and Schultz 2003; Carland and Nelson 2004; Koizumi et al. 2005; Sieburth et al. 2006; Naramoto et al. 2009), vascular tissue fragmentation is related mainly to higher-order veins, whereas the primary vein retains its continuity and a connection with the stem vascular system, which indicates the possibility of an alternative formation pathway. This other potential mechanism of vascular tissue formation may overlap with those associated with PAT-dependent vascularization and may further complicate understanding of this process. Determination of whether there are additional mechanisms responsible for vascular development is the next important challenge for future research.

#### Author contribution statement

AB contributed conception and design of manuscript; MB collected and processed the data; AB and MB wrote the chapters of manuscript, read and approved the submitted version.
